# Enhancement of mesenchymal stem cell chondrogenesis with short-term low intensity pulsed electromagnetic fields

**DOI:** 10.1038/s41598-017-09892-w

**Published:** 2017-08-25

**Authors:** Dinesh Parate, Alfredo Franco-Obregón, Jürg Fröhlich, Christian Beyer, Azlina A. Abbas, Tunku Kamarul, James H. P. Hui, Zheng Yang

**Affiliations:** 1 Department of Orthopaedic Surgery, Yong Loo Lin School of Medicine, National University of Singapore, NUHS Tower Block, Level 11, 1E Kent Ridge Road, Singapore, 119288 Singapore; 2 Department of Surgery, Yong Loo Lin School of Medicine, National University of Singapore, NUHS Tower Block, Level 8, IE Kent Ridge Road, Singapore, 119228 Singapore; 3 BioIonic Currents Electromagnetic Pulsing Systems Laboratory, BICEPS, National University of Singapore, MD6, 14 medical Drive, #14-01, Singapore, 117599 Singapore; 4 Institute for Electromagnetic Fields, Swiss Federal Institute of Technology (ETH), Rämistrasse 101, 8092 Zurich, Switzerland; 5 Tissue Engineering Group (TEG), National Orthopaedic Centre of Excellence for Research and Learning (NOCERAL), Department of Orthopaedic Surgery, Faculty of Medicine, University of Malaya, Pantai Valley, Kuala Lumpur, 50603 Malaysia; 6 Tissue Engineering Program, Life Sciences Institute, National University of Singapore, DSO (Kent Ridge) Building, #04-01, 27 Medical Drive, Singapore, 117510 Singapore

## Abstract

Pulse electromagnetic fields (PEMFs) have been shown to recruit calcium-signaling cascades common to chondrogenesis. Here we document the effects of specified PEMF parameters over mesenchymal stem cells (MSC) chondrogenic differentiation. MSCs undergoing chondrogenesis are preferentially responsive to an electromagnetic efficacy window defined by field amplitude, duration and frequency of exposure. Contrary to conventional practice of administering prolonged and repetitive exposures to PEMFs, optimal chondrogenic outcome is achieved in response to brief (10 minutes), low intensity (2 mT) exposure to 6 ms bursts of magnetic pulses, at 15 Hz, administered only once at the onset of chondrogenic induction. By contrast, repeated exposures diminished chondrogenic outcome and could be attributed to calcium entry after the initial induction. Transient receptor potential (TRP) channels appear to mediate these aspects of PEMF stimulation, serving as a conduit for extracellular calcium. Preventing calcium entry during the repeated PEMF exposure with the co-administration of EGTA or TRP channel antagonists precluded the inhibition of differentiation. This study highlights the intricacies of calcium homeostasis during early chondrogenesis and the constraints that are placed on PEMF-based therapeutic strategies aimed at promoting MSC chondrogenesis. The demonstrated efficacy of our optimized PEMF regimens has clear clinical implications for future regenerative strategies for cartilage.

## Introduction

Articular cartilage is an avascular tissue with low potential for self-repair. When left untreated, lesions of the articular cartilage can lead to osteoarthritis^1–3^. The success of any technology aimed at repairing chondral defects will thus be based on its ability to produce tissues that most closely recapitulate the mechanical and biochemical properties of native cartilage. To this end many technologies have been advanced yet, none are without drawbacks. The ‘microfracture’ technique is commonly plagued by the formation of fibro-cartilaginous tissue of low dexterity^4^. Autologous chondrocytes implantation and osteochondral autograft transplantation are limited by scarce cartilage production, low proliferative capacity of chondrocytes, chondrocyte de-differentiation and complications due to donor site morbidity^5^. Stem cell-based approaches are also being actively pursued in hopes of improved outcome. Mesenchymal stem cells (MSCs) support chondrogenic differentiation and are an attractive cell source for cartilage tissue engineering. However, the neocartilage formed by conventional MSC-based repair methodologies commonly contain a mixture of fibro- and hyaline cartilage^6–8^ that do not achieve the biochemical, mechanical or functional properties of native cartilage.

MSCs can be differentiated along different cell lineages of mesodermal origin including osteoblasts, chondrocytes, skeletal myocytes or visceral stromal cells^9^. Chondrogenic induction of MSCs entails proliferation, condensation, differentiation and maturation^10^, necessitating endogenous transcriptional and developmental regulators, cell-cell and cell-matrix interactions that, in turn, are modulated by environmental stimuli including mechanical forces, temperature and oxygen levels^10–13^. A common objective is to recreate as closely as possible the *in vivo* environmental conditions *in vitro* so that the rate and quality of chondrogenic development is enhanced and the functionality of the repaired tissue improved. To this end, various environmental stimuli such as hypoxia, mechanical, electric and electromagnetic stimulation are currently being explored^14–16^.

Mechanical stimulation can be applied in a semi-controlled manner with the use of bioreactors designed to impart shear, compression, tension, or pressure on developing tissues. Appropriately applied mechanical stimulation positively influences MSC-induced chondrogenic differentiation, ECM deposition and the mechanical properties of the generated cartilage^17–20^. At the cellular level the transduction of mechanical signals (mechanotransduction) involves their conversion into biochemical responses, often with the assistance of mechanosensitive calcium channels^21–24^. Electromagnetic field (EMF)-stimulation has been shown to promote cell differentiation via the modulation of extracellular calcium entry via plasma membrane-embedded cation channels^25–27^, raising the intriguing possibility that EMFs may be recruiting related pathways.

Studies examining time-variant or pulsing electromagnetic fields (PEMFs) have alluded to a benefit over articular chondrocytes or cartilaginous tissue *in vitro*, particularly with reference to chondrocyte proliferation, extracellular matrix (ECM) deposition, secretory activity and inflammatory status^28–35^. Studies have also examined the effects of PEMF-treatment over the chondrogenic differentiation of stem cells derived from bone marrow^36–38^, adipose^35^, umbilical cord Wharton jelly^39^, synovial fluid^40^ or peripheral blood sources^40^. The reported consequences of PEMF-stimulation over chondrogenesis, however, are largely inconsistent. Some studies report modest enhancements in the gene expression of Sox9, aggrecan, type II collagen (Col 2) as well as deposition of sulfated glycosaminoglycan (sGAG), typically on the order of 2-folds^35, 36^, whereas other studies show little to no effect^38–41^. On the extreme end of the spectrum, Wang *et al*.^37^ reported inhibition of both Sox9 and Col 2 expression concomitant with induction of hypertrophy and mineralization in response to exposures of 3 h per day at an amplitude of 1 mT. Obvious differences in stimulation protocols likely underlie reported discrepancies. Existing EMF studies have typically employed exposure durations between 30 minutes to 8 h per day and more consistently in the low milli Tesla amplitude range (3–5 mT). Empirical determination of the appropriate exposure and signal parameters for a specific biological response and given tissue are essential as there are indications that cell responses to magnetic fields obey an electromagnetic efficacy window defined by a specific combination of frequency, amplitude and time of exposure that gives rise to optimum cell response^42, 43^. Here, we systematically characterized the effects of PEMF exposure over MSC chondrogenic differentiation by varying the field amplitude, exposure duration and dosage with an emphasis on determining the briefest and lowest amplitude electromagnetic exposure to render a developmental outcome. Given that both mechanical stimuli and calcium entry^21, 22^ influences chondrogenic differentiation, we investigated the ability of PEMF exposure to influence calcium homeostasis during early induction of MSCs into the chondrogenic lineage, in particular that attributed to the Transient Receptor Potential (TRP) family of cation-permeable channels, which has been broadly implicated in cellular mechanotransduction^23, 44^. We show that brief and single exposures to low amplitude PEMFs were most effective at stimulating MSC chondrogenesis. Our results also implicate the involvement of calcium influx and the mechanosensitive TRP channels, TRPC1 and TRPV4, in the chondrogenic development stimulated by targeted PEMF exposure.

## Results

### Effect of PEMF intensities and exposure durations on MSC chondrogenesis

**Figure 1:**
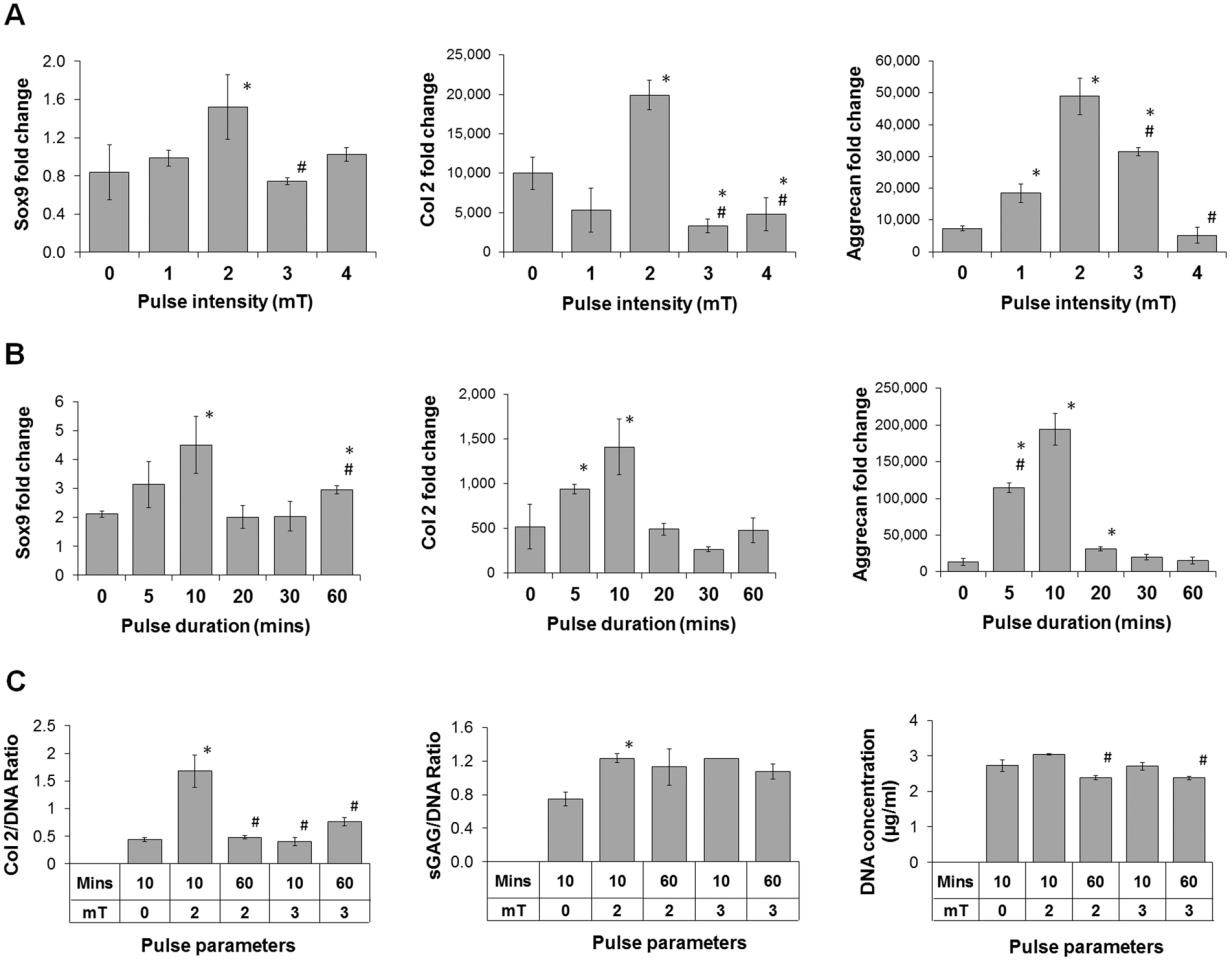
Effects of PEMF amplitude (**A**) and exposure duration (**B**) on MSC chondrogenesis. Real-time PCR analysis of cartilaginous markers expression after 7 days of differentiation was normalized to GAPDH and presented as fold-changes relative to levels in undifferentiated MSC. (**C**) Quantification of cartilaginous extracellular matrix macromolecules (Col 2 and sGAG) generated after 21 days of chondrogenic differentiation of MSC subjected to distinct PEMF parameters. All data shown are mean ± SD, n = 6 from 2 independent experiments. *Denotes significant increase, or decrease, compared to non-PEMF control. ^#^Denotes significant decrease compared to 2 mT (**A**), or 10 min (**B**) PEMF exposure.

We first sought to determine the magnetic field amplitude and duration of exposure at which MSCs undergoing chondrogenic induction are most responsive using starting conditions preliminarily tested in MSCs for chondrogenic regeneration^39^. MSC pellets in chondrogenic differentiation medium were subjected to single exposures to PEMFs of 10 min duration at intensities ranging in amplitudes between 0–4 mT (Fig. 1A), then subjected to exposure durations between 5 and 60 min at 2 mT intensity (Fig. 1B), applied on the first day of chondrogenic induction. RNA analysis monitoring MSC chondrogenic progression at 7 days post-induction showed greatest increases in response to 10 min exposures applied at an amplitude of 2 mT as evidenced by enhancements in Sox9, aggrecan and Col 2 mRNA expression. By contrast, lower (1 mT) or higher (>3 mT) amplitude of PEMFs (Fig. 1A), or briefer (5 min) or longer (>20 min) durations of exposure (Fig. 1B), resulted in overall smaller effect sizes. The same EMF efficacy window translated to the expression of cartilaginous ECM macromolecular proteins (Fig. 1C). In response to 2 mT amplitude pulsing, a 3-fold increase in Col 2 protein was detected 21 days after chondrogenic induction, whereas no increase was detected with exposure to 3 mT. Moreover, a 2-fold increase in sGAG was detected in response to exposure to 2 mT PEMFs, whereas 3 mT PEMFs produced a significantly smaller increase. The relative ineffectiveness of prolonged exposure to PEMFs was also corroborated at the protein level. Sixty min exposures to 2 mT PEMFs did not elicit significant increases in Col 2 formation than 10 min exposures (Fig. 1C). With reference to sGAG production, PEMF amplitudes greater than 2 mT, or exposure durations of one hour, produced inferior results to 10 min exposures at 2 mT.

### Dosage effects of PEMFs over MSC chondrogenesis

**Figure 2:**
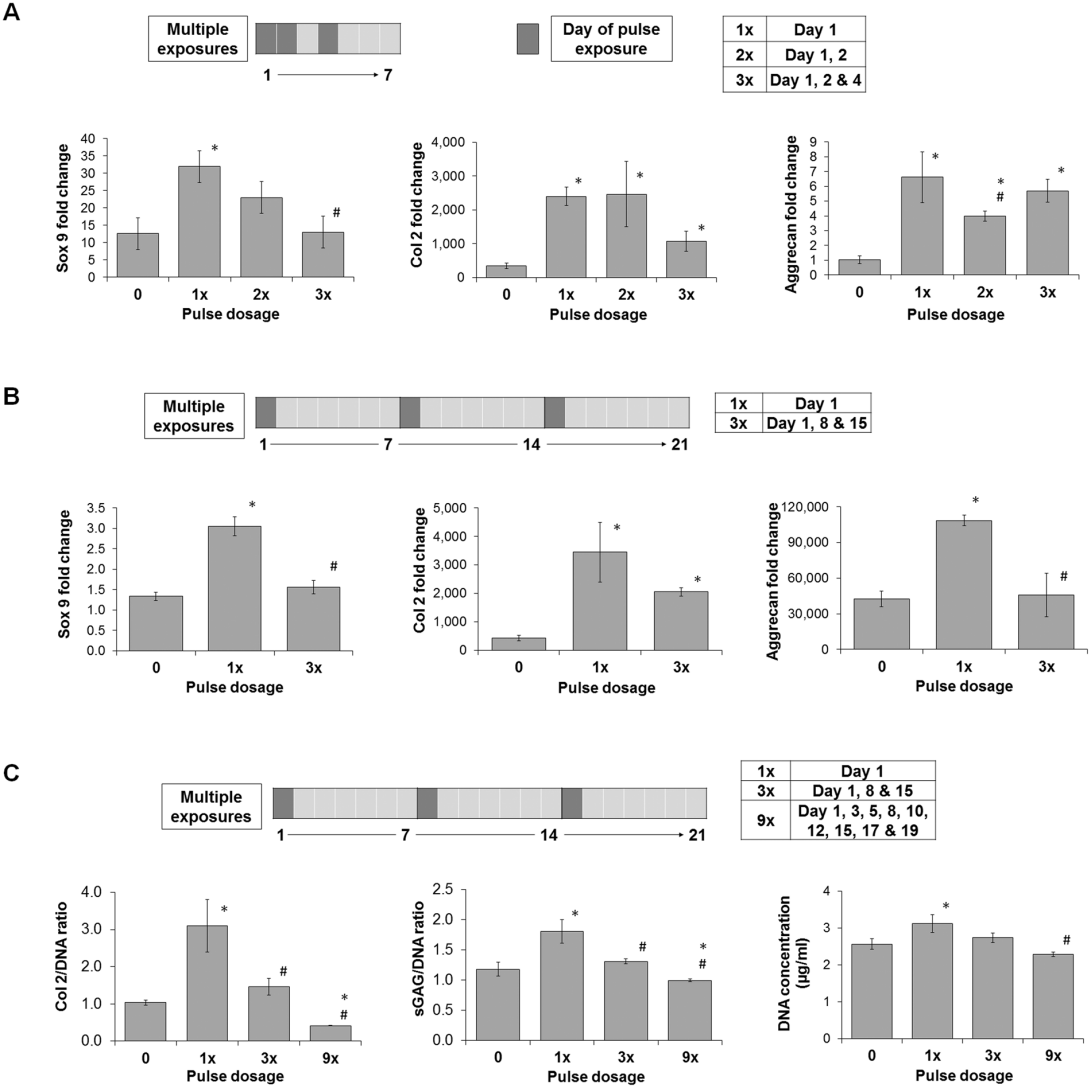
Dosage effects of PEMFs over MSC chondrogenesis. (**A**) MSCs were exposed once (1x), twice (2x) or thrice (3x) per week. (**B**) MSCs were subjected to either a single exposure on day 1 of chondrogenic induction (1x) or once per week for 3 weeks (3x). Real-time PCR analysis of cartilaginous markers expression at 7 (**A**) or 21 days (**B**) after the induction of differentiation was normalized to GAPDH and presented as fold-changes relative to level in undifferentiated MSCs. (**C**) Quantification of cartilaginous ECM macromolecules generated during chondrogenic differentiation of MSCs in response to distinct PEMF dosing as indicated. MSC pellets were subjected to either a single PEMF exposure given on day 1 of chondrogenic induction (1x) once per week for 3 weeks (3x), or thrice weekly for 3 weeks (9x). Data represents the mean ± SD, n = 6 from 2 independent experiments. *Denotes significant increase compare to non-PEMF (0 mT) control. ^#^Denotes significant decrease compared to single PEMF (1x) exposure.

We next investigated the effect of repetitive exposures to PEMFs. MSCs were exposed to PEMFs at 2 mT for 10 min/day once, twice or thrice on days 1, 2 and 4 following chondrogenic induction (Fig. 2A). RNA analysis after 7 days of differentiation showed that a single exposure produced the greatest and most consistent increase in the expression of chondrogenic markers (Fig. 2A). In another series of experiments, MSCs pellets were exposed once on the first day of chondrogenic induction, or weekly for 3 consecutive weeks (Fig. 2B). RNA analysis after 21 days of chondrogenic differentiation showed that a single exposure to 2 mT PEMFs for 10 min given on day 1 of induction gave the greatest and most consistent increase in expression of chondrogenic markers relative to no exposure (0 mT) (Fig. 2B). Three weekly exposures either rendered no additional benefit (Col 2) or gave similar results to control (0 mT) (Sox9 and aggrecan). Moreover, the amount of ECM produced was inversely related to the total number of exposures. Single exposures produced >2-folds and ~1-fold increases in Col 2 and sGAG, respectively (Fig. 2C), whereas triple weekly exposures for three weeks (9 total exposures) completely precluded an increase Col 2 and sGAG formation. The change in the amount of DNA across samples varied less than 0.2-fold, although reaching significances at 2 mT, indicating that cell proliferation was only modestly affected within our pellet culture system (Fig. 2C).

### Effect of PEMF treatment to deposition of ECM

**Figure 3:**
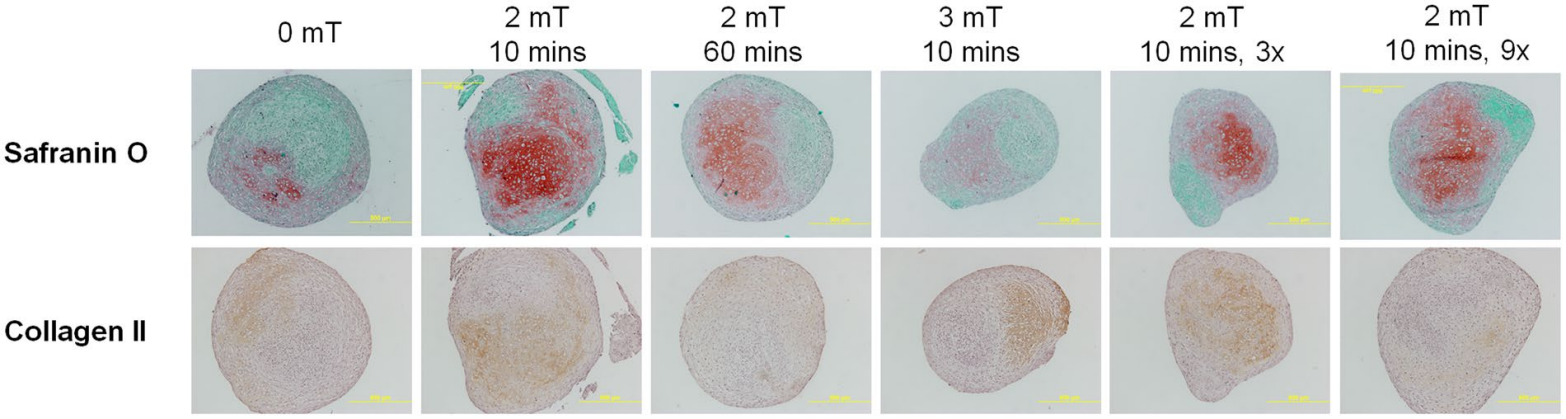
Histological analysis of pellets exposed to PEMFs of distinct amplitude, duration and dosage. Pellets were harvested at day 21, sectioned and subjected to Safranin O or type II collagen immunohistochemistry staining. Images presented were represenation of n = 3, taken at 100× magnification.

ECM deposition in response to PEMF-exposure was also analyzed using Safranin O staining for proteoglycan and immunohistochemical staining for type II collagen (Fig. 3). Stained images of day 21 samples showed an enhanced deposition of proteoglycan and type II collagen in samples exposed only once to 2 mT for 10 min as compared to control (0 mT). By contrast, MSC samples exposed for longer (60 min), to greater amplitude (3 mT) or repeatedly (3x, 9x) yielded comparable, or inferior, ECM deposition to control.

### Ca^2+^ entry pathways implicated in transducing the effects of PEMFs over MSC differentiation

**Figure 4:**
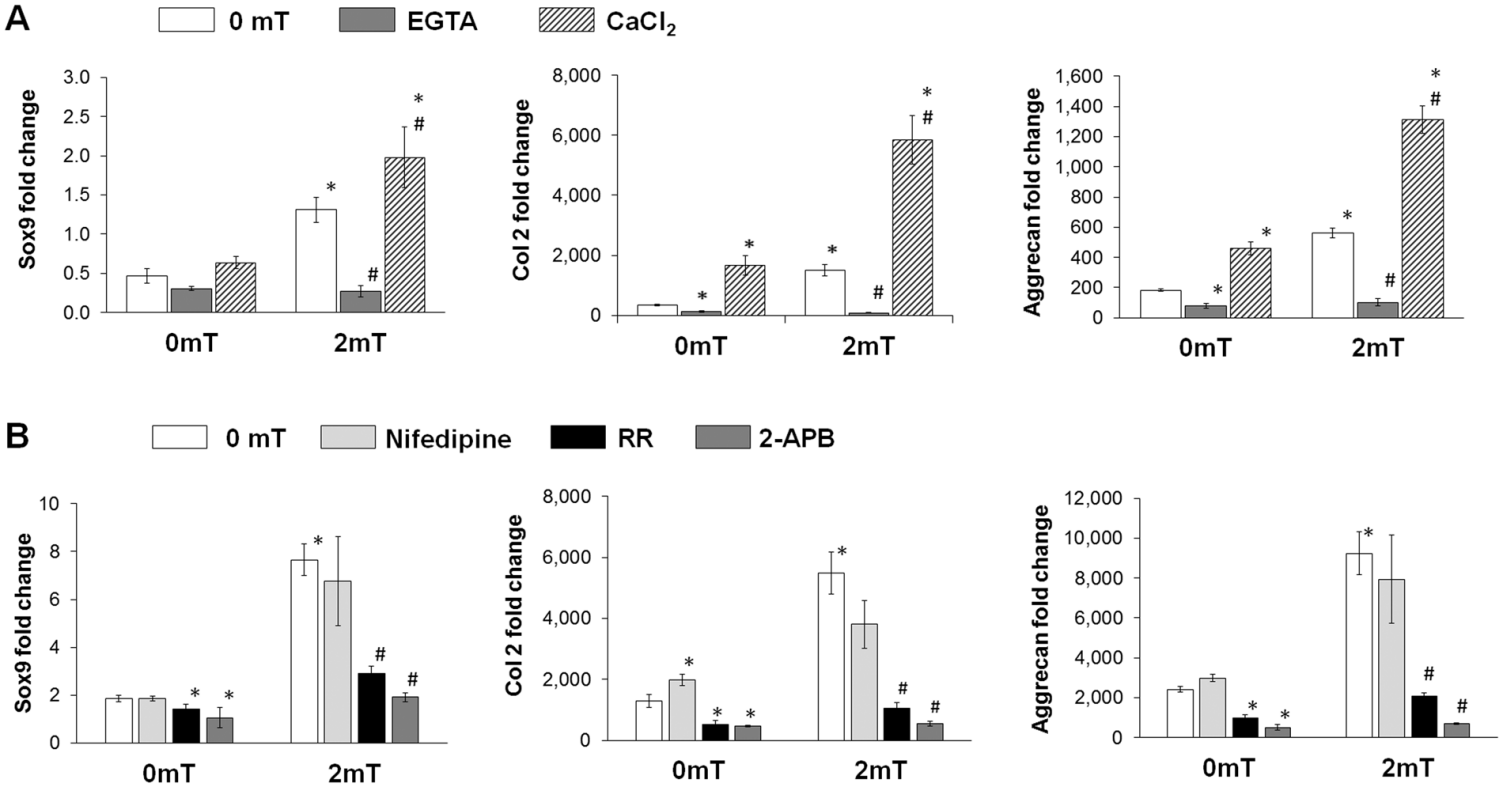
Investigation of calcium entry pathways implicated in the PEMF-effect. (**A**) Involvement of Ca^2+^ influx in mediating the effects of PEMF-induced MSC chondrogenic differentiation. MSCs were exposed for 10 min at 2 mT alone (control, white bars), or in the presence of 2 mM EGTA (dark grey bars) or 5 mM CaCl_2_ (hatched bars) transiently added to the culture media. EGTA and CaCl_2_ were included to the bathing media 10 min before exposure and replaced with age-matched media control cultures 10 min after exposure. (**B**) Involvement of candidate calcium channels in mediating the effect of PEMs over MSC chondrogenic differentiation. Control MSC chondrogenic differentiation medium (white bars) was supplemented with Nifedipine (1 µM, light grey bars), Ruthenium Red (RR, 10 µM, black bars), or 2-APB (100 µM, dark grey bars) 10 min before exposure and replaced with age-matched media control cultures 10 min after exposure. Real-time PCR analysis was performed on day 7 of differentiation. Data represent the means ± SD, n = 6 from 2 independent experiments. *Denotes significant increase, or decrease, compared to non-PEMF (0 mT) control. ^#^Denotes significant decrease relativeto 2 mT PEMF treatment.

To investigate whether PEMF-stimulated MSC chondrogenesis was depended on calcium influx, EGTA (2 mM) was co-administered to the culture medium during PEMF exposure and summarily replaced afterwards with age-matched chondrogenic control media. RNA analysis at day 7 showed that the inclusion of EGTA significantly decreased the mRNA expression of Sox9, Col 2 and aggrecan in PEMF-treated samples (Fig. 4A), indicating that PEMF-exposure stimulates calcium influx. Conversely, transiently supplementing the differentiation medium with elevated extracellular Ca^2+^ (5 mM CaCl_2_) enhanced the mRNA expression of Sox9, Col 2 and aggrecan in otherwise non-exposed samples, and moreover, accentuated chondrogenic gene expression in PEMF-treated samples. These results corroborate that calcium influx is part of the upstream signalling cascade recruited by PEMFs contributing to chondrogenic induction.

To reveal the Ca^2+^ influx pathway recruited by PEMFs, we pharmacologically dissected the contribution of candidate channels utilizing 2-APB (100 µM) or Ruthenium Red (RR, 10 µM) as TRPC or TRPV cation channel antagonists, respectively, or Nifedipine (1 µM), as a dihydropyridine-sensitive, L-type voltage-gated calcium channel (VGCC) antagonist. Calcium channel antagonists were included into the differentiation medium 10 min before exposure to PEMFs and removed immediately afterwards with age-matched control chondrogenic media. Both 2-APB and Ruthenium Red completely inhibited the PEMF-triggered up-regulation of chondrogenic genes, whereas Nifedipine had no significant inhibitory effect (Fig. 4B). Chondrogenic inhibition by 2-APB and Ruthenium Red was also observed in non-exposed samples, indicating that TRPC- and TRPV-mediated calcium entry are similarly involved in constitutive chondrogenesis upon induction. By contrast, VGCC-mediated Ca^2+^ entry does not appear to play a predominant role in the early induction of chondrogenesis.

**Figure 5:**
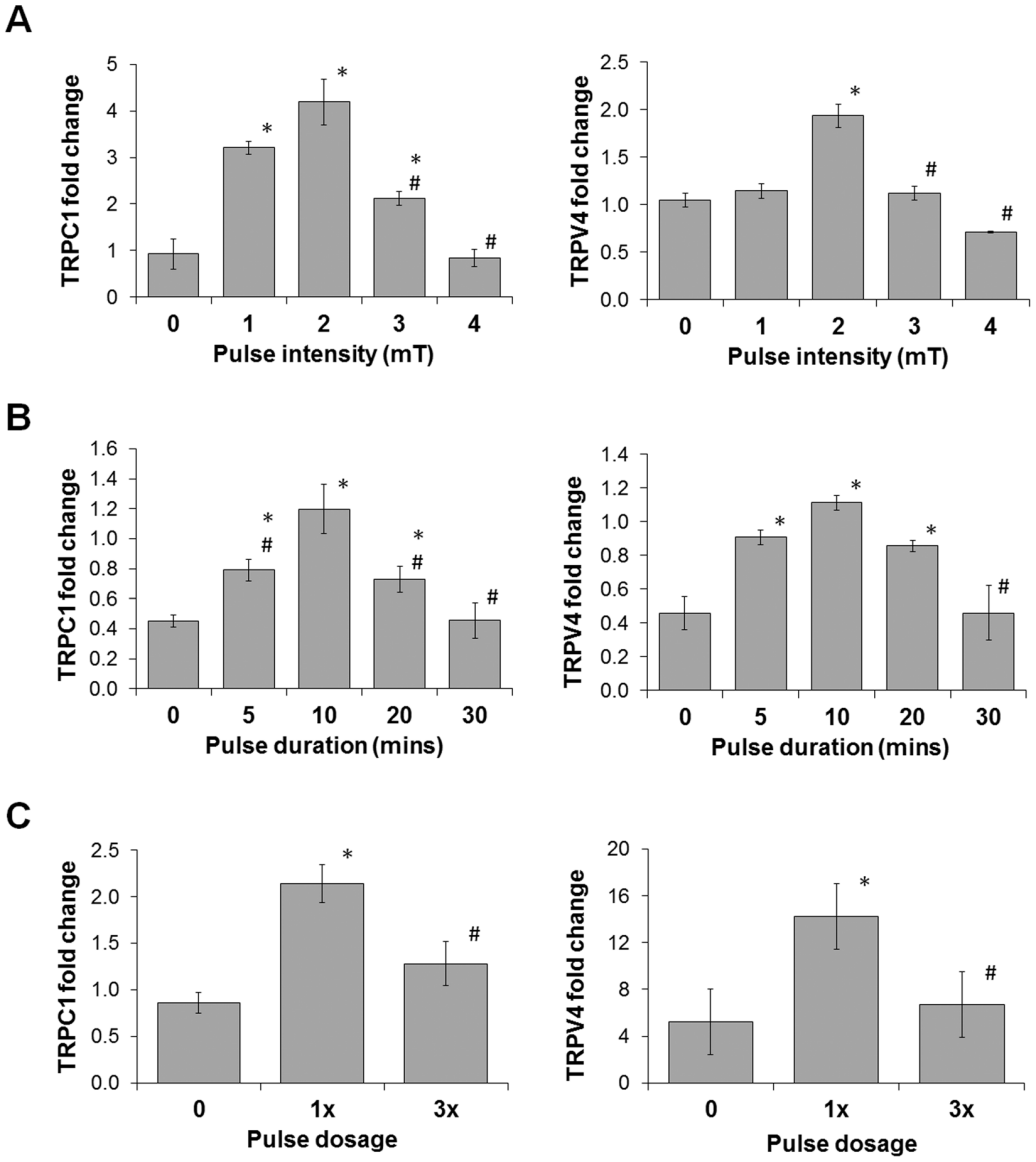
Expression profiles of TRPC1 and TRPV4 in response to determined PEMF efficacy window regulating MSC chondrogenesis. Real-time PCR analysis of TRPC1 and V4 exposed to different (**A**) intensities, (**B**) durations, and (**C**) dosages of PEMFs. Data represent the means ± SD, n = 6 from 2 independent experiments. *Denotes significant increase, or decrease, compared to non-PEMF (0 mT) control. ^#^Denotes significant decrease relative to 2 mT (**A**), 10 min (**B**), or single (1x, **C**) PEMF treatment.

We next investigated the expression profiles of TRP channels (TRPC1, TRPC6, TRPV1, TRPV4, TRPV6) previously implicated in chondrogenesis and correlated these to our PEMF-induced chondrogenic responses. Amongst the panel of candidate TRP channels, the expression of TRPC1 and TRPV4 most closely correlated with our delineated magnetic efficacy window governing chondrogenesis with reference to PEMF amplitude, duration and dosage (Fig. 5 and Suppl. Figures 1 and 2). These results corroborate an involvement of TRPC1 and TRPV4 in the PEMF-induced enhancement of chondrogenic differentiation of MSC we observed.

### Effect of recurring calcium influx on MSCchondrogenesis

**Figure 6:**
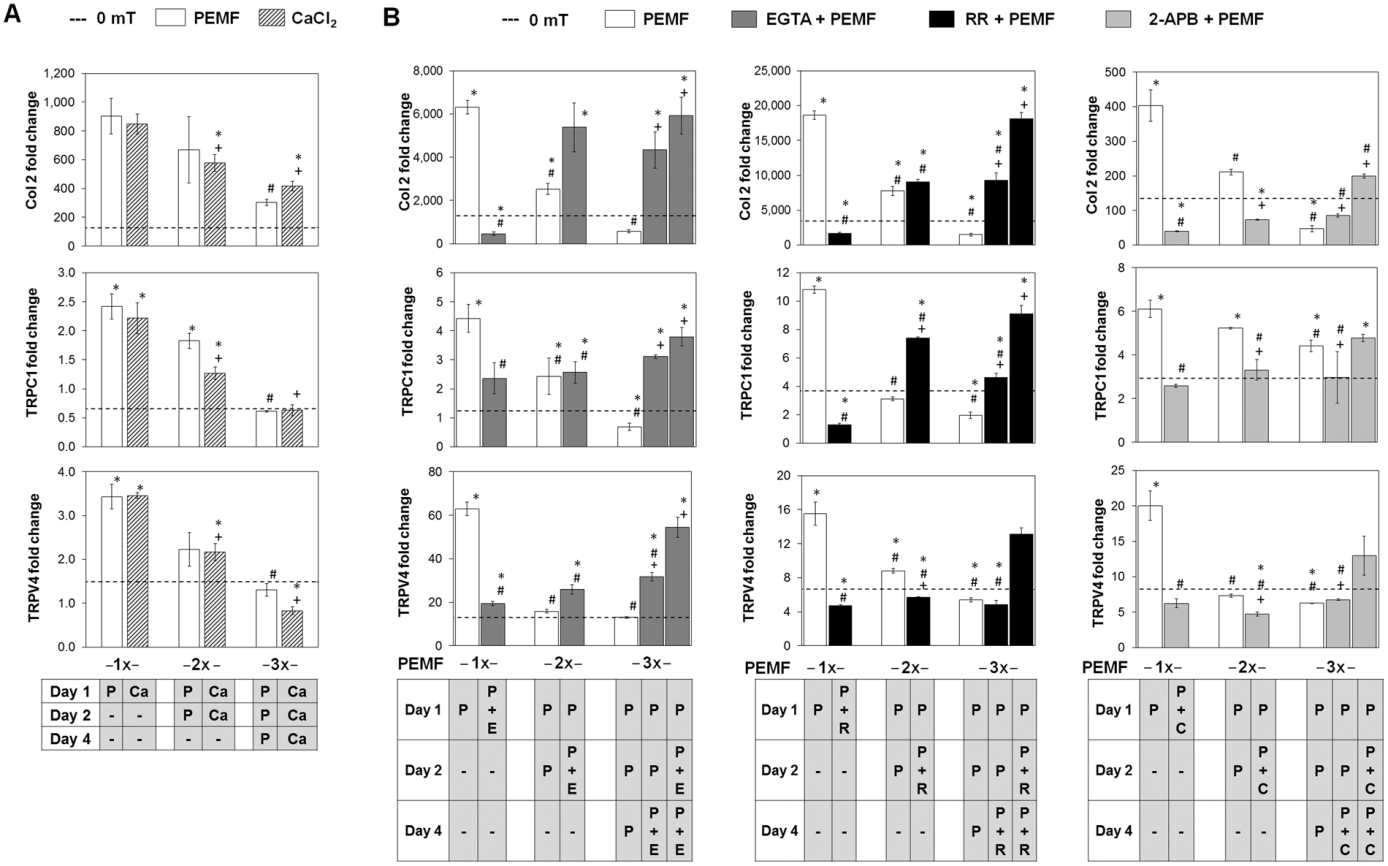
(**A**) MSC chondrogenic differentiation in response to multiple exposures to PEMFs or exogenously elevated calcium. MSCs were subjected to either PEMF stimulation alone (white bars) or with transient supplementation of CaCl_2_ alone (5 mM; hatched bars), once (1x), twice (2x) or thrice (3x) in a week. Dotted lines refers to expression level of non-treated controls. Real-time PCR analysis was performed on day 7 of chondrogenic differentiation. *Denotes significant increase relative to non-PEMF (0 mT) control. ^#^ and ^+^ denote significant differences relative to respective single (1x) exposure (white and hatched bars, respectively). P = PEMF treatment, Ca = CaCl_2_ supplementation. (**B**) MSC chondrogenic differentiation in response to multiple exposures to PEMFs alone (white bars) or in combination with calcium chelator (EGTA) or TRP channel antagonists. EGTA (2 mM; dark grey bars; “E”), Ruthenium Red (10 µM; RR, black bars; “R”) or 2-APB (100 µM; light grey bars; “C”) was added to the MSC differentiation medium during PEMF expoure applied once (1x), twice (2) or thrice (3x) per week. EGTA, RR and 2-APB were included 10 min before exposure and replaced with media harvested from age-matched chondrogenic control cultures 10 min after exposure. *Denotes significant increase compare to non-PEMF (0 mT) control. ^#^Denotes significant decrease compared to single PEMF exposure (1x). ^+^Denotes significant difference compared to respective PEMF control (white bar). P = PEMF treatment, E = EGTA, R = Ruthenium Red (RR), C = 2-APB. Data shown are means ± SD, n = 6 from 2 independent experiments.

We next investigated whether calcium entry, particularly that via TRPchannels underlies the inhibitory effect observed with repeated PEMF exposures. MSCs were exposed once, twice or thrice to 2 mT PEMFs for 10 min or, alternatively, exposed for 10 min to aged-matched control differentiation media containing elevated extracellular calcium (5 mM CaCl_2_) in lieu of PEMF exposure. RNA analysis at day 7 showed that MSCs treated once with PEMFs, or transiently administered elevated calcium, on day 1 exhibited enhanced chondrogenesis to comparable levels. By contrast, subsequent exposures to elevated calcium, on days 2 and 4 suppressed chondrogenesis mirrored the effect of multiple exposures to PEMFs (Fig. 6A and Suppl. Fig. 3A).

**Figure 7:**
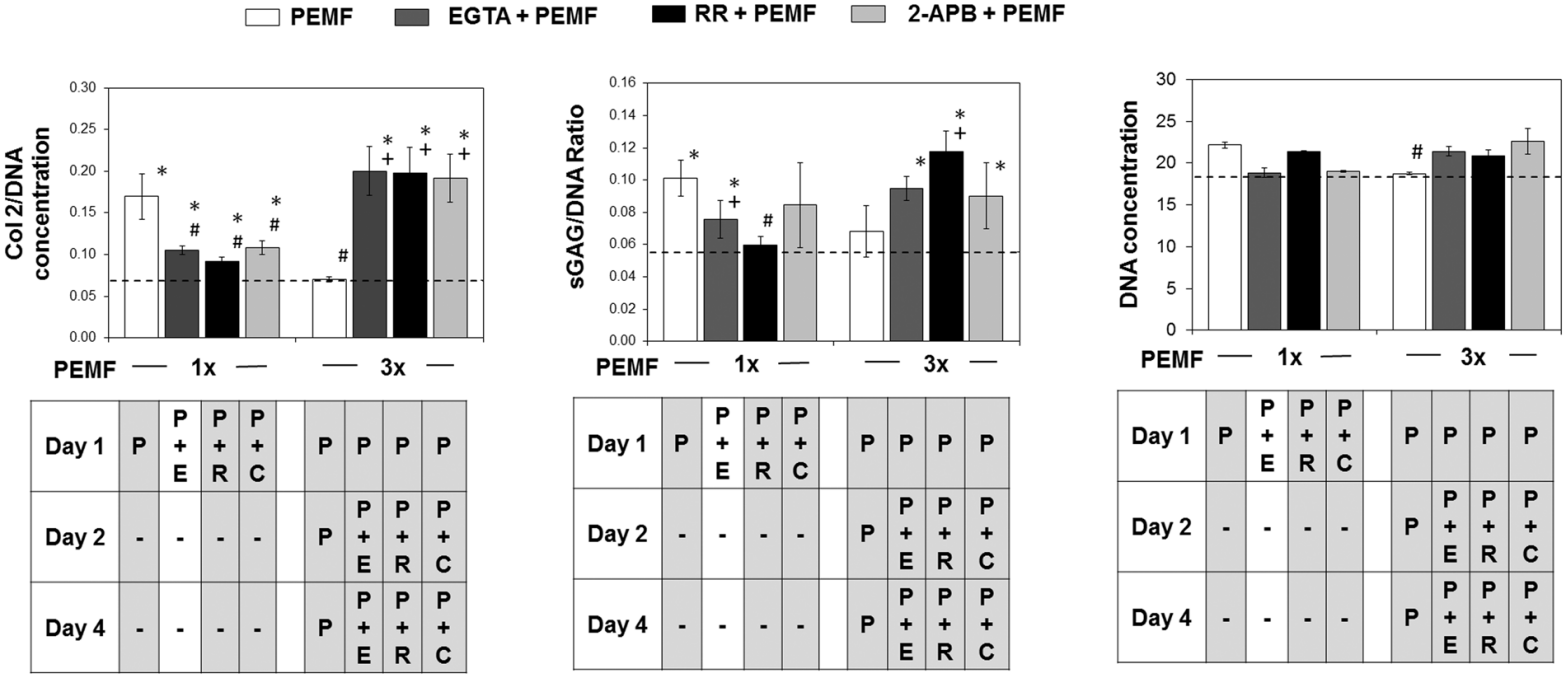
Quantification of cartilaginous ECM macromolecules generated by chondrogenically differentiated MSC in response to single or three weekly PEMF exposures alone (white bars) or in combination with calcium chelator (EGTA) or TRP channel antagonists as indicated. EGTA (2 mM; dark grey bars; “E”), Ruthenium Red (10 µM; RR, black bars; “R”) and 2-APB (100 µM; light grey bars; “C”) were included once during single PEMF exposures, or twice during the second and third PEMF exposure. EGTA, RR and 2-APB were added 10 min before exposure and replaced with media harvested from age-matched chondrogenic control cultures 10 min after exposure. *Denotes significant increase compare to non-PEMF (0 mT) control. ^#^Denotes significant decrease compared to single PEMF exposure (1x). ^+^Denotes significant difference compared to respective PEMF control (white bar). P = PEMF treatment, E = EGTA, R = Ruthenium Red (RR), C = 2-APB. Data represent means ± SD, n = 3.

Analogously, precluding calcium entry (with EGTA) also exhibited dichotomous effects if applied during the first versus the second or third exposition to PEMFs, although in opposite direction to that observed with calcium administration or PEMFs. Whereas EGTA added during the initial exposure to PEMFs (1x) prevented PEMF-induced chondrogenesis, EGTA applied during the second or third exposure partially counteracted the inhibition of differentiation exerted by serial PEMF exposure (Fig. 6B and Suppl. Fig. 3B). Notably, impeding calcium entry with transient application of EGTA during both the second and third PEMF exposure was capable of almost completely reversing the inhibition of chondrogenesis observed with repeated PEMF exposures, suggesting that PEMFs are activating disparately functioning calcium mechanisms at early (day 1) and later stages (>day 2) of chondrogenic-induction that confer opposite effects over chondrogenesis. In contrast to the beneficial effect of calcium influx induced by PEMF at the initial stage of chondrogenesis, subsequent induction of calcium influx by repeated pulsing at later stages of chondrogenesis was suppressive of MSC chondrogenesis. The contribution of TRPC- and TRPV-mediated calcium entry to the chondrogenic-inhibition observed with repeated PEMF exposures was investigated by co-administering 2-APB (100 µM) or Ruthenium Red (RR, 10 µM), respectively, during PEMF exposure. As observed with transient EGTA application, antagonism of TRPC1/V4-mediated calcium entry during the first exposition to PEMFs was strongly inhibitory of PEMF-induced chondrogenesis, whereasTRPC1/V4 antagonism during subsequent PEMF expositions was somewhat less protective than EGTA over differentiation (Fig. 6B), implicating other yet to be determined calcium pathways in the later calcium-dependent inhibitory phase of chondrogenic progression. Notably, Ruthenium Red (TRPV4 antagonist) was capable of reverting the inhibition of differentiation and expression of TRPC1 expression in response to repeated PEMFing, whereas 2-APB (TRPC antagonist) was unable to revert the inhibition of differentiation and TRPV4 expression in response to repeated PEMFing, suggesting that TRPV4-mediated calcium entry antagonizes TRPC1 expression leading up to differentiation suppression. The dichotomous effects of precluding calcium entry by EGTA, Ruthenium Red or 2-APB when applied during the first, or the second and third, exposition to PEMFs was corroborated at the protein level. Preventing calcium entry during the initial exposure to PEMFs (1x) prevented PEMF-induced cartilaginous Col 2 and sGAG formation, while blocking calcium entry at later exposures counteracted the inhibition of differentiation exerted by serial PEMF exposure (Fig. 7). Voltage-gated L-type calcium channels, on the other hand, do not appear to be strongly implicated in the response as the expression of its subunits (CACNA1C and CACNA2D1) was not perturbed by PEMF or calcium treatment (Suppl. Fig. 3).

## Discussion

Pulsed electromagnetic fields (PEMFs) have been demonstrated to be influential in numerous biological functions including progenitor cell fate determination and differentiation. PEMF-based therapies have been previously shown to enhance chondrocyte and cartilage explant anabolism while also limiting the catabolic consequences of inflammatory cytokines^29–31, 33–35, 40^. PEMF exposure has been also reported to enhance the chondrogenic induction of stem cells^34–37, 45^. Nevertheless, inconsistent and conflicting results plague the scientific literature in this area of study, with PEMF exposures typically being applied on the order of hours per day for several days or weeks at a time. Here we report a high-efficacy of unprecedentedly brief (10 min applied once) PEMF exposure at inducing MSC chondrogenesis. We consistently detected increases in Sox9, Col 2 and aggrecan mRNA (>2-folds) in response to lone exposure to 2 mT PEMFs applied at the commencement of induction for only 10 min (Fig. 1). These increments in mRNA later translated into increased chondrogenic ECM protein formation (>2-fold) after 21 days of differentiation. By contrast, stimulation with greater amplitudes (>3 mT), longer exposures (>20 min) or more frequently (>2x/week) rendered no additional benefit, or was even less effective at promoting chondrogenesis at both the gene and protein levels. Although higher PEMF amplitudes and longer duration exposures were capable of augmenting aggrecan mRNA expression and macromolecular sGAG formation, the levels achieved were no better than those from samples treated only once with 2 mT PEMFs for 10 min. Col 2 expression was especially susceptible to overstimulation, being negatively impacted by exposures >2 mT or longer than 10 minutes. To the best of our knowledge, all published EMF studies examining chondrogenesis have employed exposure durations between 30 min to 8 h^28, 35, 37, 38^. For instance, Mayer-Wagner *et al*.^38^ using PEMF of 15 Hz, 5 mT, exposed MSCs undergoing chondrogenesis for 45 min every 8 h for a total of 21 days and observed less than a 2-fold increase in type II collagen expression, with no detected effect on Sox9 or aggrecan expression. Wang *et al*.^37^ using 1, 2, and 5 mT PEMFs at the frequency of 75 Hz exposed MSCs for 3 h per day for 4 weeks and instead observed a loss of cartilaginous phenotype associated with increased cartilage-specific extracellular matrix degradation in the later stage of chondrogenic differentiation.

Given that most conventional PEMF exposure paradigms employ a multiple exposure strategy and have reported positive chondrogenic outcome^35^, we sought to determine the minimal number of exposures necessary to promote chondrogenesis (Fig. 2). We found that exposing MSCs once at the commencement of chondrogenic differentiation (1x) was necessary and sufficient to induce chondrogenic gene expression (Fig. 2A), which was sustainable for up to 21 days post chondrogenic-induction (Fig. 2B). The superior effect of a single pulse was also confirmed at the level of sGAG and Col 2 protein deposition (Figs 2C and 3). Indeed, in response to 3 exposures per week (10 min pulsing) for 3 consecutive weeks (9x treatments), ECM deposition was unchanged, or even inhibited, relative to unexposed samples. Ours is likely the first report to demonstrate an effectiveness of lone, 10 min, low amplitude PEMF exposures over MSC chondrogenesis, while concomitantly demonstrating the counter productivity of prolonged or repeated exposures. The possibility that prolonged or repeated PEMF exposures were merely cytotoxic, rather than truly inhibitory to chondrogenesis, was ruled out by our finding that total DNA content across all treatments was largely unchanged, despite lower Col 2 yield. In addition, the amount of sGAG was either unchanged or higher than that in control non-pulsed samples, further indicating that prolonged/repeated PEMF exposure did not adversely influence cell viability. Finally, the PEMF paradigm demonstrated here to best promote chondrogenesis (2 mT applied once for 10 min) did not alter the expression of osteogenic genes, Runx2 and ALP (Suppl. Fig. 4). Provocatively, osteogenic markers did increase following 20 min exposure to 2 mT PEMFs, thereby substantiating our assertion that reduced chondrogenic expression is not a reflection of cell death, but likely deferred chondrogenesis towards osteogenesis. Our demonstration of the high efficacy of brief and early PEMF exposure might thus help explain the existing inconsistencies and the relatively weaker responses previously reported^28, 30, 32, 34–38^.

Chondrogenesis is known to be modulated by calcium signaling cascades of specific temporal sensitivity^24, 46^. The dependence of chondrogenesis on extracellular Ca^2+^ was first alluded to with the demonstration that elevated extracellular Ca^2+^ promoted chondrogenic differentiation in chick limb bud-derived cultures^47, 48^. Moreover, Sox9, the master transcription factor of chondrogenesis, is subject to Ca^2+^-calmodulin regulation^49^. Elevation in cytoplasmic calcium downstream of calcium influx has been demonstrated in response to electric field (EF) or EMF stimulation during MSC-derived osteogenesis or chondrogenesis^27, 50, 51^. We show that MSC chondrogenesis depends on the presence of extracellular Ca^2+^, whereby a transient (10 min) elevation of extracellular Ca^2+^ or brief (10 min) exposure to PEMFs (Figs 4A and 6A) enhanced MSC chondrogenic differentiation in an additive manner. Previous studies have also revealed that chondrogenesis is positively responsive to intracellular Ca^2+^ within a tightly controlled concentration window^24, 46^. A 1.25-fold increase in cytosolic Ca^2+^ concentration was shown to promote differentiation, whereas a moderately greater increase (1.5-fold) negatively influenced *in vitro* chondrogenesis^48^. It is thus feasible that high amplitude or prolonged PEMF exposures elevate cytoplasmic calcium levels beyond the beneficial threshold for MSC chondrogenic differentiation^46^. It is also well documented that the spatial and temporal patterns of intracellular free Ca^2+^ concentration play important roles in the regulation of various cellular processes, governed not only by absolute Ca^2+^ level, but also by periodic oscillatory changes of cytosolic Ca^2+^ concentration^48, 52^. MSCs undergoing chondrogenesis increase their frequency of Ca^2+^oscillations (waves) in the early stages of differentiation^48^, coinciding with the initial period of cellular condensation during the first 2–4 days^10^. Conversely, sustained elevations of extracellular calcium inhibit chondrogenesis^48^, demonstrating a temporal requirement for calcium. Here we show that transient pulsing with elevated calcium recapitulates the temporal characteristic of the inhibitory actions of repeated PEMF exposures (Fig. 6A). Moreover, preventing calcium entry (with EGTA) during repeated PEMF exposure precludes the inhibition (Figs 6B and 7), defining a developmental change in calcium-sensitivity following calcium-dependent initiation of chondrogenesis. In this respect, single brief exposition to PEMFs defined by a specified electromagnetic window applied during the early stages of MSC chondrogenesis may be sufficient to provide the correct catalytic rise in intracellular Ca^2+^ to optimally promote the initiation of chondrogenesis. Conversely, higher exposure intensities or multiple exposures could result in excessive or sustained calcium influx that may instead disrupts or interrupts MSC-induced chondrogenesis, respectively.

Ca^2+^ influx via membrane-associated cation channels is a key event in initiating chondrogenesis, that can be potentially mediated by either TRP channels and/or voltage-gated calcium channels (VGCC)^24, 44, 46^. The transient receptor potential (TRP) channels are a diverse and widely distributed family of cation channel broadly implicated in cellular mechanotransduction^23, 53–57^. The TRPC and TRPV subfamilies have been broadly implicated in calcium homeostasis, ascribed mechanically-mediated gating^44, 57–61^, as well as implicated in the developmental programs of diverse mechanosensitive tissues^62, 63^. Previous studies have shown that blocking TRPV4 during the initial stages of induction inhibited chondrogenesis^60, 64^. TRPV4-mediated Ca^2+^ signaling is also a positive regulator of Sox9 and as such, has been shown to promote chondrogenesis^65^ and in transducing the mechanical signals that support cartilage extracellular matrix maintenance and joint health^44, 59^. TRPC1 is expressed during early chondrocyte expansion^66^, as well as being involved in the proliferation of mesenchymal stem cells^67^. We detected time, intensity, and PEMF dosage-dependent up-regulations of both TRPV4 and TRPC1 that closely correlated with the PEMF-induced expression pattern of chondrogenic markers (Fig. 5). Blocking TRPC1 and TRPV4 channels with 2-APB^68^ and Ruthenium Red^64^, respectively, in the early stage of differentiation effectively inhibited chondrogenesis, implicating these TRP channels in the initiation of chondrogenesis, and indicating that PEMFs recruit the activity of these channels to enhance chondrogenesis. Notably, blocking calcium-permeation through TRPV4 channels reverses the inhibition on chondrogenic differentiation and TRPC1 expression during repeated PEMF exposure, whereas blocking TRPC1 channels was unable to revert the inhibition on differentiation and expression of TRPV4 in response to repeated PEMF exposure, suggesting that TRPV4-mediated calcium entry antagonizes TRPC1 expression and is an essential step in initiating differentiation (Figs 6B and 7). TRPV4-mediated calcium entry may thus increase after the induction of differentiation (>2 days) serving to curtail TRPC1 expression and thereby promote differentiation by inhibiting TRPC1-medited proliferation (Suppl. Fig. 2).

An involvement of voltage-gated calcium channels was more difficult to establish. A predominant role for L-type VGCCs (CACNA1, CACNA2D1) in transducing PEMF’s effects was not supported given that a chondrogenically-effective dose of, Nifedipine, a L-type VGCC antagonist^53–55^, had no significant effect on the PEMF-induced upregulation of MSC chondrogenesis (Fig. 4B). Moreover, the expression level of the L-type channel was not correlated with changes in calcium (Suppl. Fig. 3). Ca^2+^ influx via the low-threshold T-type VGCC had been previously implicated in tracheal chondrogenesis^56^. The expression of T-type VGCC (CACNA1H) was induced by lone early exposure to PEMFs or transient calcium administration, and was suppressed by repeated exposures to PEMF or extracellular calcium, mirroring the expression pattern of chondrogenic markers under identical conditions (Suppl. Fig. 3A). The induction of the T-type calcium channel in response to PEMF/calcium exposure more likely reflects chondrogenic differentiation, rather than a fully determinant role in PEMF-induced chondrogenesis, as its expression was in the majority of conditions unchanged (relative to control) by removal of extracellular calcium during PEMF exposure (Suppl. Fig. 3B). Our strongest data support the interpretation that TRPC1 and TRPV4 play a more predominant role, although not necessarily exclusive, in transducing the chondrogenic effects of PEMFs. Further work will require to fully disentangle the intricasy of calcium homeostasis during the chondrogenic developmental process.

In summary, we have provided comprehensive characterization of the effects of PEMFs over MSC chondrogenic differentiation. MSCs undergoing chondrogenic induction are preferentially responsive to a well-defined window of PEMF stimulation of particular amplitude (2 mT), duration (10 min) and dosage (once on day 1 induction). By contrast, treatment with higher amplitude PEMFs, longer exposure durations or repeated expositions, as are more common in the field, are generally counterproductive, helping explain the lack of resolution in the field. Our results indicate that PEMFs mediate their effect by activating calcium influx through mechanosensitive calcium TRP channels. The unprecedented efficacy of our low amplitude, exceptionally brief and non-invasive PEMF-exposure protocol over MSC chondrogenesis has broad clinical and practical implications for the ultimate translation of related PEMF-based therapeutic strategies for stem cell-based cartilage regeneration.

## Methods

### Human bone marrow MSCs culture and chondrogenic differentiation

Primary human mesenchymal stem cells (MSCs) were purchased from RoosterBio Inc. (Frederick, MD), supplied at passage 3. The MSCs was further expanded in MSC High Performance Media (RoosterBio Inc.) at 37 °C in 5% CO_2_ atmosphere. The expanded MSCs were used at passage 5–6. Chondrogenic differentiation of MSCs was induced through 3D pellet culture as previously described^16, 69^. Briefly, 2.5 × 10^5^ cells were centrifuged to form pellets and cultured in a chondrogenic differentiation medium containing high glucose DMEM supplemented with 4 mM proline, 50 µg/mL ascorbic acid, 1% ITS-Premix (Becton-Dickinson, San Jose, CA), 1 mM sodium pyruvate, and 10^−7^ M dexamethasone (Sigma-Aldrich, St Louis, MO), in the absence of antibiotics, for up to 7 or 21 days in the presence of 10 ng/mL of transforming growth factor-β3 (TGFβ3; R&D Systems, Minneapolis, MN). To investigate an involvement of calcium influx or of calcium channels in transmitting the effects of PEMFs, cells were pre-incubated in chondrogenic media supplemented with elevated calcium, EGTA or particular calcium channel antagonist for 10 minutes prior to pulsing. Ten minutes after exposure to PEMFs the supplemented chondrogenic media was replaced with age-matched chondrogenic media (0 mT) cultures. To attenuate extracellular calcium influx, 2 mM ethylene-bis(oxyethylenenitrilo) tetraacetic acid (EGTA; Sigma) was added to the bathing media as noted. To promote extracellular Ca^2+^ influx the bathing media was supplemented with 5 mM CaCl_2_ (Sigma). To block calcium permeation through dihydropyridine-sensitive, L-type voltage-gated calcium channel (VGCC), Nifedipine (1 µM, Sigma) was added to the bathing media. 2-aminoethoxydiphenyl borate (2-APB, 100 µM, Sigma) and Ruthenium Red (10 µM, Merck Millipore) were administered as indicated to block calcium entry via TRPC and TRPV channels, respectively. Aminoglycoside antibiotics such as streptomycin were excluded in all MSC expansion and chondrogenic differentiation media to avoid interference with mechanosensitive ion channels^70^.

### PEMF Exposure system

The ELF-PEMF (extremely low frequency – pulsed magnetic field) delivery system has been described previously^43^. For the purposes of this study a barrage of magnetic pulses of 6 ms duration was applied at a repetition rate of 15 Hz and at flux densities between 1–4 mT. Each 6 ms burst consisted of a series of 20 consecutive asymmetric pulses of 150 µs on and off duration with an approximate rise time of 17 T/s. The background magnetic flux density measured in the chamber was below 1 µT between 0 Hz to 5 kHz. The coil size, position and individual number of windings were numerically optimized by a CST low frequency solver for low field non-uniformity over a wide frequency range taking into consideration the shielding capacity of the µ-metallic chassis. The measured field non-uniformity did not exceed 4% within the uniform exposure region of the coils.

### PEMF treatment

To investigate the optimum dosage of PEMF, MSCs in a 3D pellet culture were exposed to PEMFs of different exposure durations, dosage and the magnetic flux amplitude. MSCs were subjected to PEMFs of 1–4 mT amplitude with exposure times ranging between 5 to 60 min on the day of chondrogenic induction, applied once or multiple times as indicated in the respective figure legend. Cell pellets to be treated once with PEMFs (1x) were exposed on first day of chondrogenic induction. Two scenarios of multiple exposures were administrated (Fig. 2). Firstly, multiple exposures were administrated during the course of a week; double exposures (2x) were applied on days 1 and 2; triple exposures (3x) on days 1, 2 and 4. Alternatively, multiple exposures were applied on a once a week basis, for up to three week. Non-exposed (control) cells were placed within the PEMF device without current flux to produce a magnetic field to ensure that all cells were subject to the same climatic and mechanical conditions.

### Real time PCR analysis

Chondrogenic cell pellets were digested in 0.25% Type II collagenase (Gibco, Life Technologies) followed by centrifugation. Total RNA was extracted using the RNeasy® Mini Kit (Qiagen, Germany). Reverse transcription was performed with 100 ng total RNA using iScript™ cDNA synthesis kit (Bio-Rad, USA). Real-time PCR was conducted using the SYBR^®^green assay on ABI 7500 Real-Time PCR System (Applied Biosystems, Life Technologies, USA). Real-time PCR program was set at 95 °C for 10 min, followed by 40 cycles of amplifications, consisting of a 15 s denaturation at 95 °C and a 1 min extension step at 60 °C. Primer sequences used in this study were according to previous publication^16^ and presented as Supplementary Table 1. The level of expression of the target gene, normalized to GAPDH, was then calculated using the 2^−ΔΔCt^ formula with reference to the undifferentiated MSC. Results were averaged from triplicate samples of two independent experiments.

### ECM and DNA quantification

Samples harvested were digested with 10 mg/mL of pepsin in 0.05 M acetic acid at 4 °C, followed by digestion with elastase (1 mg/mL). A Blyscan sulfated glycosaminoglycan (sGAG) assay kit (Biocolor Ltd., Newtownabbey, Ireland) was used to quantify sGAG deposition according to manufacturer’s protocol. Absorbance was measured at 656 nm and sGAG concentration was extrapolated from a standard curve generated using a sGAG standard. Type II Collagen (Col 2) content was measured using a captured enzyme-linked immunosorbent assay (Chondrex, Redmond, WA). Absorbance at 490 nm was measured and the concentration of Col 2 was extrapolated from a standard curve generated using a Col 2 standard. Values for sGAG and Col 2 content obtained were normalized to the total DNA content of respective samples, measured using Picogreen dsDNA assay (Molecular Probes, OR, USA). Quadruplicates of each group were analyzed from two independent experiments.

### Histological and immunohistochemical evaluation

Samples were fixed in formalin, dehydrated, paraffin embedded, and cut into sections of 5 µm. For Safranin-O staining, the sections were incubated in hematoxylin (Sigma-Aldrich), washed and stained with fast green (Sigma-Aldrich), before staining with Safranin-O solution (AcrosOrganics). For immunohistochemistry, ultra-vision detection kit (Thermo scientific) was used. Endogenous peroxidase in the sections was first blocked with hydrogen peroxide before pepsin treatment for 20 min. Samples were treated with monoclonal antibodies of collagen type II (Clone 6B3; Chemicon Inc.) followed by incubation with biotinylated goat anti-mouse (Lab Vision Corporation). A mouse IgG isotype (Zymed Laboratories Inc.) was used as control for immunohistochemistry studies.

### Statistical analysis

All experiments were performed in biological replicates (n = 3 or 4) and results reported as mean ± standard deviation (SD). Statistical analysis was carried out by Students t-test for comparison between two groups using the Microsoft Excel software. The level of significance was set at p < 0.05. All quantitative data reported here were averaged from at least two independent experiments.

## Electronic supplementary material

Supplementary Information
