# Supplementary material for: Enhancement of mesenchymal stem cell chondrogenesis with short-term low intensity pulsed electromagnetic fields
Source: Sci Rep. 2017 Aug 25;7:9421. doi: 10.1038/s41598-017-09892-w (PMC5572790; doi:10.1038/s41598-017-09892-w)
Supplement: Supplementary file 1 — Supplementary Information [file 41598_2017_9892_MOESM1_ESM.pdf]

Enhancement of mesenchymal stem cell chondrogenesis with short-term low intensity pulsed electromagnetic fields

Dinesh Parate [1], Alfredo Franco-Obregón [2,3,*], Jürg Fröhlich [2,4], Christian Beyer [4], Azlina A Abbas [5], Tunku Kamarul [5], James HP Hui [1,6,*], Zheng Yang [1,6,*]

Supplementary Information

Table 1. PCR primer sequences.

Gene	Forward Primer Sequence	Reverse Primer Sequence
GAPDH	ATGGGGAAGGTGAAGGTCG	TAAAAGCAG CCCTGGTGACC
Aggrecan	ACTTCCGCTGGTCAGATGGA	TCTCGTGCCAGATCATCACC
Sox9	AGTACCCGCACTTGCACAA	CTCGTTCAGAAGTCTCCAGAGCTT
Col II	GGCAATAGCAGGTTCACGTACA	CGATAACAGTCTTGCCCCACTT
TRPC1	TGGATGTTGCACCTGTCATT	TTACATTGCCGGGCTAGTTC
TRPV4	CTACGCTTCAGCCCTGGTCTC	GCAGTTGGTCTGGTCCTCATTG

The primer sets of L-type dihydropyridine-sensitve voltage-gated calcium channel, CACNA1 and CACNA2D1, and T-type low threshold voltage-gated calcium channel, CACNA1H, were from Biorad (USA).

Supplementary Figure 1

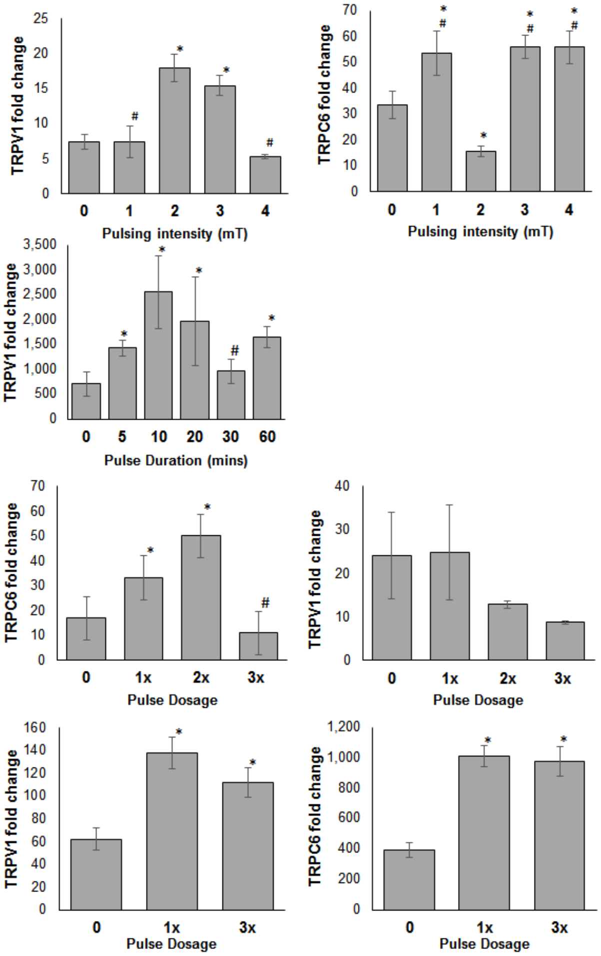

Suppl. Fig. 1. TRP channel expression in chondrogenically differentiated MSC pellets in response to PEMF treatment. Real-time PCR analysis of TRPV1 and C6 exposed to A) different amplitudes, B) duration of exposure, and C) dosage of PEMFs. Data represent the means ± SD, n=6 from 2 independent experiments. * denotes significant increase, or decrease, compared to non-PEMF (0 mT) control. $^{\#}$ denotes significant decrease compared to 2mT PEMF treatment.

Supplementary Figure 2

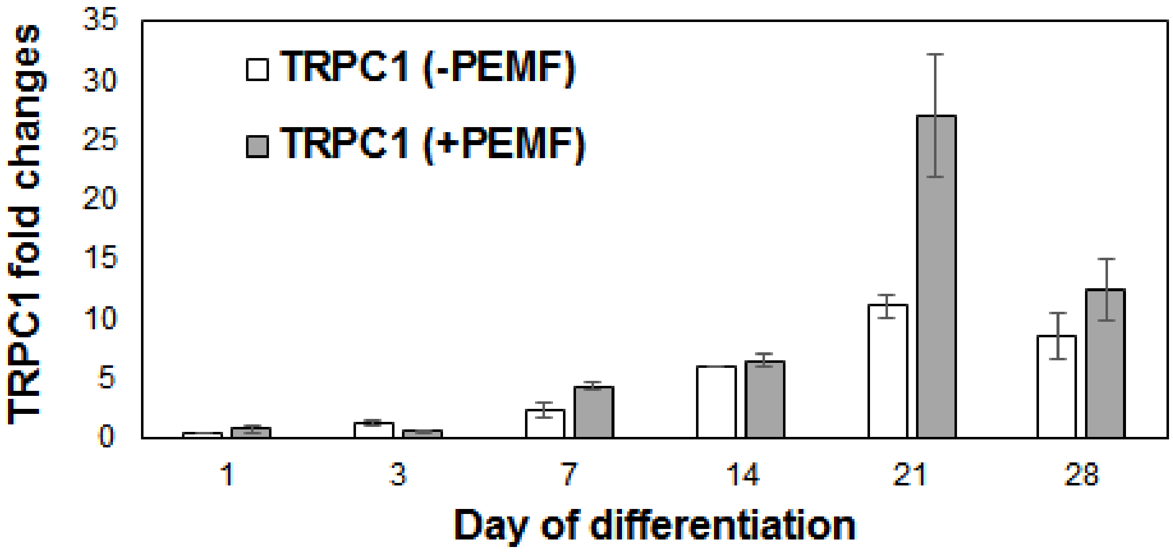

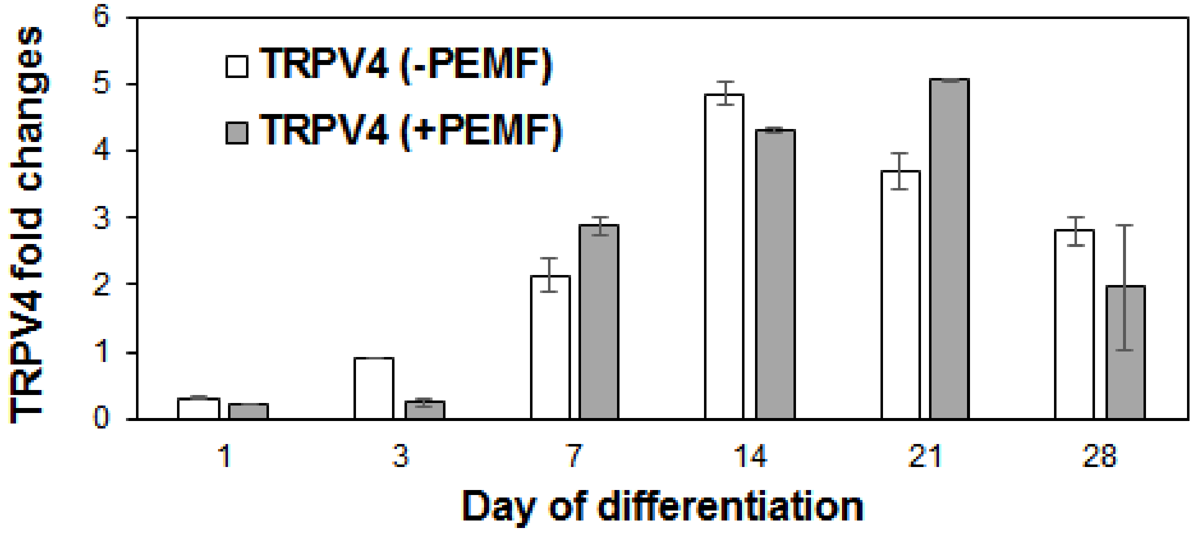

Suppl. Fig 2. Developmental time course of TRPC1 and TRPV4 gene expression during chondrogenic induction (at day 1) with and without PEMF stimulation. TRPC1 expression is strongly upregulated with PEMF stimulation following chondrogenic induction.

Supplementary Figure 3

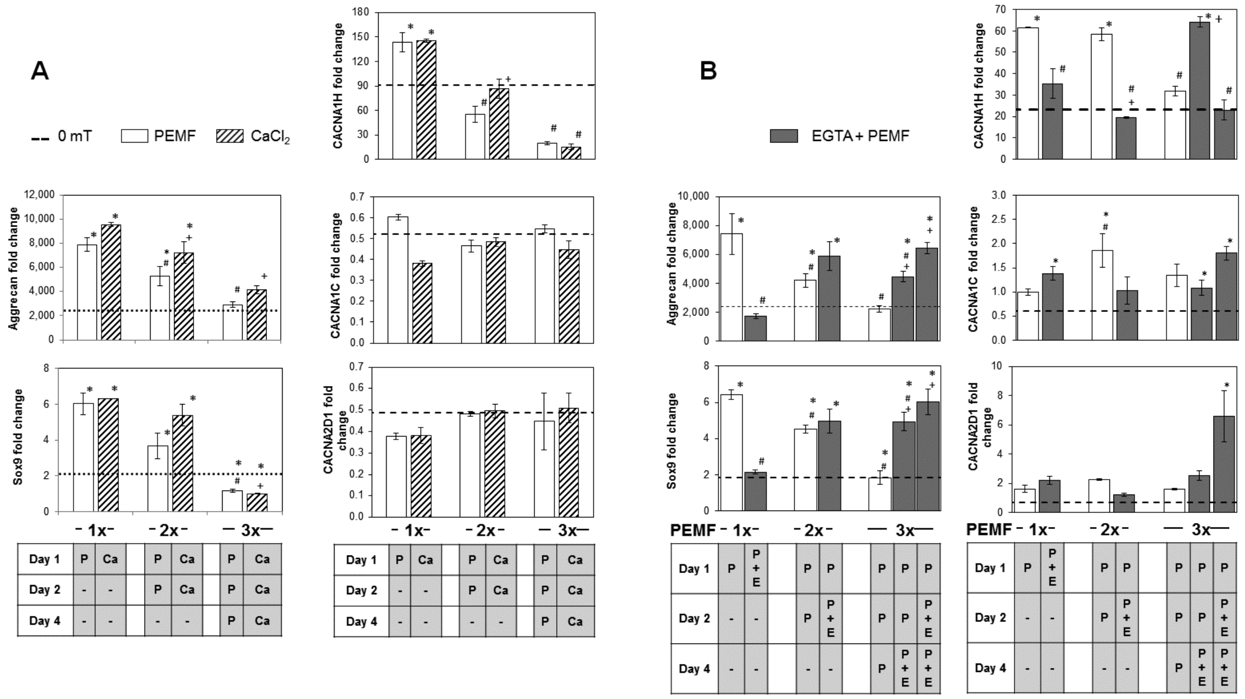

Suppl. Fig 3. (**A**) MSC chondrogenic differentiation and voltage-gated calcium channel (VGCC) expression in response to repeated PEMF exposure or exogenously elevated extracellular calcium (5 mM). MSCs were either subjected to either PEMF stimulation (white bars) or transient administration of elevated extracellular $CaCl_2$ (5 mM; hatched bars), once (1x), twice (2x) or thrice (3x) in a week. Elevated extracellular calcium was included 10 min before pulse, and replaced with media harvested from age-matched chondrogenic control cultures 10 min after exposure. Data represents means ± SD, n=6 from 2 independent experiments. * denotes significant increase relative to non-PEMF (0 mT) control. $^{\#}$ and + denotes significant difference relative to its respective single PEMF dose (1x) (white and grey bars, respectively). P = PEMF treatment, Ca = $CaCl_2$ supplementation. (**B**) Effect of extracellular calcium chelation over PEMF-induced MSC chondrogenic differentiation and VGCC expression. MSC differentiation medium was supplemented with (dark grey bars) or without (white bars) 2 mM EGTA during the PEMF stimulation applied once (1x), twice (2) or thrice (3x). EGTA was included 10 min before pulse, and replaced with media harvested from age-matched chondrogenic control cultures 10 min after exposure. Dotted lines refer to expression level in non-treated controls. Data shown are means ± SD, n=6 from 2 independent experiments. * denotes significant increase compare to non-PEMF (0 mT) control. $^{\#}$ denotes significant decrease compared to single pulse (1x) treatment. $^{+}$ denotes significant difference compared to respective PEMF control (white bar). P = PEMF treatment and E = EGTA supplementation. CACNA1: L-type dihydropyridine-sensitve voltage-gated calcium channel; CACNA2D1: L-type dihydropyridine-sensitve voltage-gated calcium channel; CACNA1H: T-type low threshold voltage-gated calcium channel; Chondrogenic genes as previously discussed.

Supplementary Figure 4

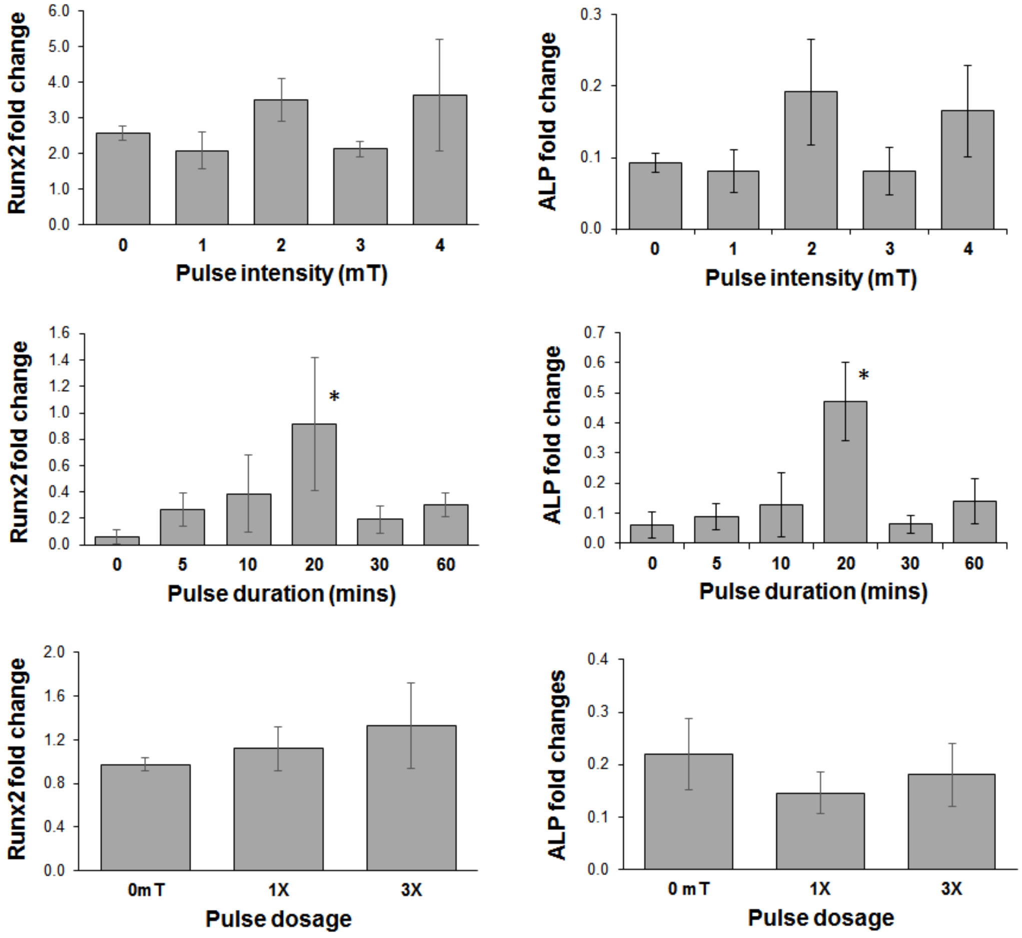

Suppl. Fig 4. Osteogenic gene (Runx2 and ALP) expression in MSC pellets undergoing chondrogenic differentiation in response to different PEMF intensities (at 10 min), duration (at 2 mT) and dosage (at 2 mT for 10 min).
